# Colorectal cancer and associated genetic, lifestyle, cigarette, nargileh-hookah use and alcohol consumption risk factors: a comprehensive case-control study

**DOI:** 10.3389/or.2024.1449709

**Published:** 2024-10-11

**Authors:** Abdulbari Bener, Ahmet Emin Öztürk, Muhammed Furkan Dasdelen, Cem Cahit Barisik, Zehra Betul Dasdelen, Ahmet F. Agan, Jean De La Rosette, Andrew S. Day

**Affiliations:** ^1^ Department of Biostatistics and Public Health, School of Medicine, Istanbul Medipol University, İstanbul, Türkiye; ^2^ Department of Evidence for Population Health Unit, School of Epidemiology and Health Sciences, The University of Manchester, Manchester, United Kingdom; ^3^ Department of Oncology, Prof. Dr. Cemil Tascioglu City Hospital, University of Health Science, Istanbul, Türkiye; ^4^ International School of Medicine, Istanbul Medipol University, Istanbul, Türkiye; ^5^ Department of Radiology and Pathology, School of Medicine, Istanbul Medipol University, Istanbul, Türkiye; ^6^ Department of Medicine, GI Unit, School of Medicine, Istanbul Medipol University, Istanbul, Türkiye; ^7^ Department of Urology, International School of Medicine, Istanbul Medipol University, Istanbul, Türkiye; ^8^ Department of Pediatrics, University of Otago Christchurch, Christchurch, New Zealand

**Keywords:** epidemiology, colon cancer, hereditary, lifestyle, dietary, cigarette, hookah, alcohol

## Abstract

**Aim:**

This study aimed to investigate the causes and risk factors of colorectal cancer (CRC) in a Turkish population, focusing on various modifiable and non-modifiable risk factors.

**Methods:**

A hospital-based case-control design was employed to compare individuals with CRC (cases) to individuals without CRC (controls). Male and female participants were recruited from the surgery, internal medicine, and out-patient departments. The study encompassed socio-demographic data, clinical information, radiological diagnoses, and biochemical measurements. Univariable and multivariable logistic regressions were used to determine associated risk factors of CRC.

**Results:**

The study included 704 individuals with CRC and 704 controls. Significant socio-demographic disparities were observed between the groups, with over 30% of the cases having lower levels of education and income compared to the controls. Lifestyle factors such as obesity, higher rates of smoking (cigarettes and hookah) and alcohol consumption were more prevalent among cases than controls. Further significant associations were identified with intestinal inflammation, obesity, processed food consumption, and symptoms such as abdominal pain, cramps, diarrhea, constipation, blood in stool, bloating, irritable bowel syndrome, nausea/vomiting, anemia, stress, fatigue, weakness, and weight loss. Diet analysis revealed that individuals with CRC consumed more red meat, processed and fast foods along with less pulses and vegetables. Genetic predispositions and exposure to chemicals also correlated strongly with increased CRC risk. Multivariable regression analysis identified, nausea/vomiting, constipation, intestinal disease, genetics factor, hookah-nargileh use, history of any cancer, family history of bowel cancer, constipation, cigarette smoking, stress, milk-yogurt consumption, obesity and red meat consumption as significant determinants for CRC.

**Conclusion:**

CRC risk is influenced by dietary, lifestyle, and genetic factors. Awareness of hereditary risk and participation in screening are crucial. Lifestyle changes, such as avoiding smoking, hookah, and alcohol use, and adopting a healthy diet, are essential for prevention.

## 1 Introduction

Colorectal cancer (CRC) is the 3rd most common cancer worldwide with 1.9 million new cases and was the second most fatal cancer with a death toll about 1 million in 2020 (1–3). Moreover, the incidence of CRC is on the rise in low- and middle-income countries for both men and women (1). This geographic variation has been mainly attributed to differences in diet, lifestyle, genetics, and environmental factors (4–6). The highest incidence rates for CRC are observed in developed countries, where they maintain a stable trend, meanwhile, rates have increased rapidly in developing countries (5–7), likely due to the adoption of Western lifestyles and the rise in early-onset CRC, defined as CRC occurring before the age of 50, which has been documented globally (2–8).

Previous studies have focused on hereditary syndromes, genetic factors, and modifiable environmental risk factors, including obesity, sedentary lifestyle, poor diets, alcohol consumption, and smoking, which are currently major problems in low- and middle-income countries (4–11). Therefore, the trend and burden of CRC is moving from developed countries to low and middle-income countries.

Gastrointestinal (GI) cancers, which include stomach, pancreatic, and colorectal cancers, account for more than a quarter of all cancer cases (12, 13). Smoking and alcohol use are well-known risk factors for cancer, and there is a growing consensus that dietary habits are also crucial in CRC development. Numerous comprehensive studies strongly support the role of diet in early-onset CRC, in particular (14–18).

Modifiable risk factors play an important role in the development of CRC (4, 5, 14–18). Evidence from several studies indicates that the risk of CRC increases with specific dietary patterns, consumption of dairy products, meat, diets low in vegetables, smoking (including e-cigarettes and hookah/water-pipe use), high body mass index (BMI), alcohol use, physical inactivity, and sedentary lifestyles (5, 6). Moreover, in recent years, rates of CRC have begun to stabilize and decline, largely due to targeted intervention and screening programs (19, 20). Thus, addressing these modifiable risk factors and screening programs could potentially reduce CRC incidence.

The aim of this study was to investigate the causes and risk factors of colon cancer, including genetics, age, chemical exposure, obesity, poor diet, lifestyle habits, cigarette and hookah smoking, alcohol use, and intestinal inflammations, as well as the consumption of processed foods to identify critical CRC risk factors.

## 2 Subjects and methods

### 2.1 Participants

The study based on cases and control design involving participants aged above 35 years who sought medical care at the Oncology and Gastrointestinal clinics, as well as out-patient clinics, at Istanbul Medipol University Hospitals and Prof. Dr. Cemil Taşcıoğlu and SSK Okmeydanı Hospitals between 24 April 2023, and 10 June 2024.

In our case-control study, the inclusion criteria for the control group involved individuals who matched the age and gender distribution of the cases within the specified data collection time and were sourced from primary healthcare facilities, internal medicine departments, and gastrointestinal (GI) clinics. Importantly, these individuals had no history of colorectal cancer (CRC) and had not undergone major surgeries affecting gastrointestinal function. The exclusion criteria for both cases and controls were stringent to maintain data integrity: subjects were excluded if they lacked complete radiological test results or comprehensive biochemistry lab analysis results. Subjects who had recently undergone medical procedures potentially impacting gastrointestinal health were also excluded, ensuring that all participants’ current health status accurately reflected their long-term condition without acute medical interventions skewing the results. CRC cases were confirmed through diagnostic methods including Magnetic Resonance Imaging (MRI), ultrasound, PET/CT, and colonoscopy. In total, 141 CRC patients and 100 control patients were excluded due to lack of follow-up or incomplete lab results.

The study adhered to the principles outlined in the Declaration of Helsinki (1964) and obtained ethical clearance and approval from the ethics committee (RP# and IRB# E-10840098-772.02-2645). The determination of the sample size was based on the anticipated proportion in controls (p = 0.05), an assumed odds ratio (OR = 2), a confidence interval (CI = 0.99), and a power of 0.80. The total calculated sample size was 1,506 with a 1:1 ratio.

The study encompassed a detailed questionnaire focusing on diverse socio-demographic factors, including age, gender, family history, BMI, smoking and hookah-nargileh use, alcohol consume, milk-yogurt consume, chemical exposure (long-term exposure to toxic metals), physical activity, intestinal inflammations (inflammatory bowel diseases), functional gastrointestinal symptoms (abdominal pain and cramps, constipation, diarrhea), and pre-existing health conditions. Content validity, face validity and reliability of the questionnaire were based on a convenience sample of 100 patients and the test revealed a high level of validity and high degree of repeatability (kappa = 0.84).

Additionally, patients’ last biochemical test results such as full blood count, liver function tests, vitamin D levels, ferritin levels, renal function tests and electrolyte levels were recorded. The diagnosis of intestinal inflammation in relevant individuals was based on a clinical diagnosis with histological findings. Hypertension was taken according to the WHO which is Systolic Blood Pressure (SBP) ≥140 mmHg, or Diastolic Blood Pressure (DBP) ≥90 mmHg, or the use of anti-hypertensive medication.

### 2.2 Statistical analysis

The statistical analysis was performed using the R (version 4.3.3) (21) and Statistical Packages for Social Sciences (SPSS v25, IBM Corp., Armonk, NY). Student t test was used to ascertain the significance of differences between mean values of two continuous variables. Chi-square analysis was performed to test for differences in proportions of categorical variables between two or more groups. We used Leas absolute shrinkage and selection operator (LASSO) method for multivariable logistic regression analysis to find out the best predictors for diagnosis of colorectal cancer as dependent variable. Best lambda value for penalizing variables was determined by 5 folds cross-validation method. Post-hoc collinearity was checked by variance inflation factor. For p values of multiple comparisons, we applied Benjamini-Hochberg method (false discovery rate correction) to decrease Type 1 error. The level p < 0.05 was considered as the cut-off value for significance.

## 3 Results

### 3.1 Socio-demographic and lifestyle factors influencing colorectal cancer risk

This study included a total of 1,408 participants: 704 subjects with colorectal cancer (CRC) and 704 controls without CRC, consisting of both males and females aged 35-75 years. Socio-demographic characteristics of the individuals with CRC differed from those of the controls (Table 1). The cases were more commonly single with lower levels of education and income. Additionally, a larger proportion of the cases with CRC were obese compared to controls.

**TABLE 1:** Socio-demographic characteristics of cases with colorectal cancer (CRC) and controls (N = 1,408).

Variable	CRC cases (N = 704) n (%) or μ±σ	Controls (N = 704) n (%) or μ±σ	Adjusted p-values^**^
Age (in years)	62.8 ± 11.79	61.60 ± 12.94	0.073
Age groups (in years):	n (%)	n (%)	
<50	113 (16.1)	130 (18.5)	
50–64	288 (40.9)	304 (43.2)	0.197
>65 and above	303 (43.0)	270 (38.4)	
Gender			
Males	357 (50.7)	360 (51.1)	0.874
Female	347 (49.3)	344 (48.9)	
Marital status			
Single	72 (10.2)	48 (6.8)	
Married	632 (89.8)	656 (93.2)	0.044
Education			
Primary	103 (14.6)	37 (5.3)	
Secondary	119 (16.9)	106 (15.1)	
High	238 (33.8)	254 (36.1)	0.008
University	244 (34.7)	307 (43.6)	
Occupation			
Housewife	195 (27.7)	118 (16.8)	
Professional/Sedentary	227 (32.2)	258 (36.6)	
Police/Army/Security	35 (5.0)	41 (5.8)	0.004
Businessman	80 (11.4)	96 (13.6)	
Manual	167 (23.7)	191 (27.1)	
Household income:			
Low	251 (35.7)	200 (28.4)	
Medium	271 (38.5)	297 (42.2)	0.037
High	182 (25.9)	207 (29.4)	
BMI^*^ (kg/m2):			
Normal (<25 kg/m2)	183 (26.0)	206 (29.3)	
Overweight (29-30 kg/m2)	294 (41.8)	313 (44.5)	0.070
Obese (>30 kg/m2)	227 (32.2)	185 (26.3)	

* BMI, body mass index.

** p values have been corrected according to FDR correction.

Comparison was made between the food consumption and daily life habits in the cases with CRC and the controls (Table 2). The cases consumed fewer vegetables and pulses compared to controls, while their intake of red meat, processed and fast foods was higher. Additionally, higher rates of malnutrition, contraceptive pill use, smoking, hookah use, and alcohol consumption were noted among the cases. However, daily activities like walking for 30 min and sleeping less than 6 h were similar between the groups.

**TABLE 2:** Univariable analysis for identifying associations of colorectal cancer with habits, foods, medications and daily activities.

Variable	CRC cases		Control		Odds ratios [95% CI]	Adjusted p values
	Yes n (%)	No n (%)	Yes n (%)	No n (%)		
Vegetable consumption	192 (27.3)	512 (72.7)	240 (34.1)	464 (65.9)	0.73 [0.58, 0.91]	0.008
Pulse consumption	180 (25.6)	524 (74.4)	222 (31.5)	482 (68.5)	0.75 [0.59, 0.94]	0.017
Fast food consumption	197 (26.5)	547 (73.5)	156 (22.2)	548 (77.8)	1.36 [1.07, 1.74]	0.016
Processed food consumption	157 (22.3)	547 (77.7)	94 (13.4)	610 (86.6)	1.86 [1.41, 2.47]	<0.001
Eating red meat 3/week	355 (50.4)	349 (49.6)	298 (42.3)	406 (57.7)	1.39 [1.12, 1.71]	0.004
Milk/yoghurt at least glass	229 (32.5)	475 (67.5)	178 (25.3)	526 (74.7)	1.42 [1.13, 1.80]	0.005
Malnutrition	134 (19.0)	570 (81.0)	85 (12.1)	619 (87.9)	1.71 [1.28, 2.30]	0.001
Calcium supplement	308 (43.8)	396 (56.2)	250 (35.5)	454 (64.5)	1.41 [1.14, 1.75]	0.003
Taking a multivitamin 4/week	163 (23.2)	541 (76.8)	119 (16.9)	585 (83.1)	1.48 [1.14, 1.93]	0.005
Vitamin D supplement	246 (34.9)	458 (65.1)	187 (26.6)	517 (73.4)	1.48 [1.18, 1.87]	0.001
Birth control pill use	151 (43.5)	196 (56.5)	67 (19.5)	277 (80.5)	3.15 [2.24, 4.43]	<0.001
Postmenopausal pill use	154 (44.4)	193 (55.6)	64 (18.6)	280 (81.4)	3.45 [2.44, 4.88]	<0.001
15 years of aspirin use	203 (28.8)	501 (71.2)	165 (23.4)	539 (76.6)	1.32 [1.04, 1.68]	0.027
Smoking	171 (24.3)	533 (75.7)	98 (13.9)	606 (86.1)	1.98 [1.51, 2.61]	<0.001
Hookah use	138 (19.6)	566 (80.4)	73 (10.4)	631 (89.6)	2.11 [1.55, 2.86]	<0.001
Alcohol use	118 (16.8)	586 (83.2)	73 (10.4)	631 (89.6)	1.74 [1.27, 2.38]	0.001
Walking 30 min a day	185 (26.3)	519 (73.7)	174 (24.7)	530 (75.3)	1.09 [0.85, 1.38]	0.541
Physical activity (moderate or vigorous)	309 (43.9)	395 (56.1)	331 (47.0)	373 (53.0)	0.88 [0.11, 0.72]	0.268
Sleeping ≤6 h	139 (19.7)	565 (80.3)	158 (22.6)	542 (77.4)	0.84 [0.65, 1.09]	0.231

Patients with CRC had higher incidences of obesity, genetic risk factors, exposure to chemicals, previous malignancies, a family history of colon cancer, and intestinal problems compared to the healthy cohort (Table 3).

**TABLE 3:** Univariable analysis for identifying associations of colorectal cancer with previously determined risk factors.

Variable	CRC cases		Control		Odds ratios [95% CI]	Adjusted p values
	Yes n (%)	No n (%)	Yes n (%)	No n (%)		
Obesity	116 (16.5)	588 (83.5)	78 (11.1)	626 (88.9)	1.58 [1.16, 2.15]	0.005
Genetic factors	146 (20.7)	558 (79.3)	63 (8.9)	641 (91.1)	2.66 [1.94, 3.65]	<0.001
Exposure to chemicals	64 (9.1)	640 (90.9)	35 (5.0)	669 (95.0)	1.91 [1.25, 2.93]	0.005
Any cancer disease	107 (15.2)	597 (84.8)	61 (8.7)	643 (91.3)	1.89 [1.35, 2.64]	<0.001
Intestinal disease	216 (30.7)	488 (69.3)	98 (13.9)	606 (86.1)	2.74 [2.10, 3.57]	<0.001
Family history of bowel cancer	129 (18.3)	575 (81.7)	66 (9.4)	638 (90.6)	2.17 [1.58, 2.98]	<0.001

There were differences in the symptoms of functional gastrointestinal disorders (FGIDs) between the cases and controls, including abdominal pain, abdominal cramps, diarrhea, constipation, blood in the stool, bloating regularly, irritable bowel syndrome, nausea/vomiting, anemia, stress, fatigue, weakness, and weight loss (Table 4).

**TABLE 4:** Univariable analysis for identifying associations of colorectal cancer with functional gastrointestinal symptoms.

Variable	CRC cases		Control		Odds ratio [95% CI]	Adjusted p values
	Yes n (%)	No n (%)	Yes n (%)	No n (%)		
Constipation	192 (27.3)	512 (72.7)	79 (11.2)	625 (88.8)	2.97 [2.23, 3.95]	<0.001
Diarrhea	172 (24.4)	532 (75.6)	118 (16.8)	586 (83.2)	1.61 [1.24, 2.09]	0.001
Blood in the stool	134 (19.0)	570 (81.0)	82 (11.6)	622 (88.4)	1.78 [1.32, 2.40]	<0.001
Abdominal pain	166 (23.6)	538 (76.4)	87 (12.4)	617 (87.6)	2.19 [1.65, 2.91]	<0.001
Abdominal cramp	160 (22.7)	544 (77.3)	99 (14.1)	605 (85.9)	1.80 [1.36, 2.37]	<0.001
Bloating (flatulence)	181 (25.7)	523 (74.3)	117 (16.6)	587 (83.4)	1.74 [1.34, 2.25]	<0.001
Irritable bowel	129 (18.3)	575 (81.7)	66 (9.4)	638 (90.6)	2.17 [1.58, 2.98]	<0.001
Anemia	153 (21.7)	551 (78.3)	108 (15.3)	596 (84.7)	1.53 [1.17, 2.01]	0.004
Feeling fatigue	177 (25.1)	527 (74.9)	120 (17.0)	584 (83.0)	1.63 [1.26, 2.12]	<0.001
Nausea/vomiting	161 (22.9)	543 (77.1)	84 (11.9)	620 (88.1)	2.19 [1.64, 2.92]	<0.001
Stress	150 (21.3)	554 (78.7)	66 (9.4)	638 (90.6)	2.62 [1.92, 3.57]	<0.001
Weight loss	128 (18.2)	576 (81.8)	65 (9.2)	639 (90.8)	2.18 [1.59, 3.01]	<0.001

### 3.2 Multivariable analysis of risk factors associated with colorectal cancer

Multivariable regression analysis was conducted to identify the most important risk factors for patients diagnosed with CRC as the dependent variable (Table 5). According to the analysis, several factors were significantly associated with CRC.

**TABLE 5:** Associated risk factors of colorectal cancer using multivariable regression analysis^a^ (N = 1,408).

Independent variables	Regression coefficients	Standard errors	Exp (coef)	95% confidence intervals	p-values
Nausea/vomiting	0.87	0.17	2.38	1.72–3.30	<0.001
Constipation	0.66	0.17	1.93	1.39–2.70	<0.001
Stress	0.72	0.19	2.05	1.42–2.95	<0.001
Obesity	0.36	0.18	1.44	1.01–2.04	0.042
History of any cancer	0.78	0.19	2.18	1.51–3.15	<0.001
Genetic factors	0.75	0.18	2.12	1.49–3.02	<0.001
Family history of bowel cancer	0.69	0.19	1.99	1.38–2.88	<0.001
Intestinal disease	0.90	0.15	2.45	1.82–3.30	<0.001
Cigarette smoking	0.72	0.16	2.06	1.51–2.81	<0.001
Hookah use	0.78	0.18	2.18	1.54–3.09	<0.001
Red meat consumption 3 times/week	0.33	0.12	1.39	1.09–1.76	0.008
Milk-yoghurt consumption	0.39	0.13	1.47	1.13–1.90	0.004
Pulse consumption	−0.22	0.13	0.81	0.62–1.05	0.108

^a^ Adjusted for education level, income level and occupation type.

Gastrointestinal symptoms were significantly associated with CRC. Nausea/vomiting (OR = 2.38, 95% CI = 1.72–3.30, p < 0.001), intestinal disease (OR = 2.45, 95% CI = 1.82–3.30, p < 0.001), and constipation (OR = 1.93, 95% CI = 1.39–2.70, p < 0.001) were notably associated with a higher risk of CRC.

Genetic factors and history of malignancies also played significant roles. The presence of genetic factors (OR = 2.12, 95% CI = 1.49–3.02, p < 0.001), a history of any cancer (OR = 2.18, 95% CI = 1.51–3.15, p < 0.001), and a family history of bowel cancer (OR = 1.99, 95% CI = 1.38–2.88, p < 0.001) were all associated with higher risks of CRC.

Lifestyle factors, including the use of hookah (OR = 2.18, 95% CI = 1.54–3.09, p < 0.001) and cigarette smoking (OR = 2.06, 95% CI = 1.51–2.81, p < 0.001) were also associated with the development of CRC. Additionally, stress (OR = 2.05, 95% CI = 1.42–2.95, p < 0.001) and obesity (OR = 1.44, 95% CI = 1.01–2.04, p = 0.042) were important risk factors.

Dietary habits were also associated with CRC. Frequent consumption of milk and yoghurt (OR = 1.47, 95% CI = 1.13–1.90, p = 0.004) and red meat three times per week (OR = 1.39, 95% CI = 1.09–1.76, p = 0.008) were both linked to higher CRC risk.

## 4 Discussion

This study investigated the factors associated with colorectal cancer in a Turkish population. Patients were prospectively selected from those admitted to Oncology and Gastrointestinal clinics for treatment or follow-up of colorectal cancer. The control group consisted of individuals from gastrointestinal clinics who had not had colorectal cancer at any point in their lifetime. The results showed that a number of socio-economic, lifestyle, dietary and genetics factors were associated with CRC.

Colorectal cancer (CRC) is one of the most prevalent cancers worldwide (1–5). In numerous evidence-based studies dietary patterns, consumption of dairy and dairy products, meat, vegetable-poor diet, smoking and e-cigarettes, nargileh-hookah, BMI, obesity, alcohol consumption, physical inactivity and sedentary lifestyle have been well-established as risk factors for CRC (19, 20, 22–28) The current study confirmed those risk factors in Turkish population, aligning well with Harvard Cancer Risk Index findings (29).

Several studies have established a link between high intake of red and processed meats and an increased risk of colorectal cancer (CRC) (15, 16, 24, 25, 30) A recent meta-analysis has further reinforced this association, highlighting meat consumption as a major factor, and suggests that dietary interventions that reduce red and processed meat intake could significantly lower CRC incidence (23). On the other hand, diets rich in fiber and possibly most dairy products are associated with a protective effect against CRC (22, 25, 26) Additionally, recent findings suggest that white meat, such as fish and poultry, does not increase CRC risk and may even confer protective benefits (25). The interaction between intestinal microbiota and fiber intake is indicated as a possible mechanism that mediates CRC risk, underscoring the importance of high fiber in reducing this risk (26).

Although several studies have provided evidence of the impact of alcohol consumption on CRC (6, 7, 27, 28), alcohol consumption levels are very low in many Muslim countries, which has a minimal effect on colorectal cancer (CRC) rates (5–7). A pooled analysis by Archambault et al. (31) suggests that early-onset CRC may be significantly associated with heavier alcohol use.

The increasing rates of smoking, both in developing and developed countries, present a worrying trend with implications for the rising incidence of CRC (1, 2). Previous studies have shown that smoking is associated with an increased risk of colorectal cancer (CRC) and its various molecular pathological subtypes (32, 33). Additionally, smoking is linked to poorer survival outcomes. A recent study in Taiwan investigated whether cigarette smoking is associated with survival in patients with CRC through a nationwide population-based cohort study (34). This study reported that the effects of smoking were more pronounced in males with CRC. Additionally, several studies have indicated that cigarette smoking is significantly associated with poor survival in individuals with CRC (5, 6, 35, 36). In the current study, CRC rates were higher among those who smoked cigarettes and used hookahs. No study has yet been conducted to investigate the mechanism through which hookah water-pipe use impacts the risk CRC.

The prevalence of obesity has increased significantly worldwide, and this trend is likely to continue in the coming years. Substantial evidence indicates that obesity plays a crucial role in the development of CRC. Epidemiological data have consistently shown a correlation between obesity and CRC (37). Understanding that obesity is a potentially modifiable risk factor that can affect the incidence and prognosis of CRC is crucial for the prevention and treatment of the condition (17, 20, 37).

Inflammatory bowel diseases (IBD), primarily including ulcerative colitis and Crohn’s disease, involve chronic inflammation of the gastrointestinal tract. The exact causes of these diseases are not entirely known, but they are thought to occur in individuals who are genetically predisposed and exposed to certain environmental risk factors. More than 0.4% of people in Europe and North America are affected by IBD and this number is expected to increase over time (38). Aligning well with the literature, we observed that patients with IBD or intestinal diseases have a higher likelihood of developing colorectal cancer (CRC), primarily the result of chronic intestinal inflammation. Additionally, because the treatment of IBD often involves immunosuppressive therapies, these patients are also at an increased risk for both gastrointestinal and non-gastrointestinal cancers (38, 39). The treatment of CRC in patients with underlying chronic inflammatory diseases are also complex. Indeed, treatment of current neoplasms in IBD patients, as well as the treatment of IBD in patients with prior malignancies, has become a challenge with limited evidence-based data available, since patients with an active or recent history of cancer or those who develop cancer while being treated with newer therapies are usually excluded from randomized controlled trials (39). Thus, both doctors and individuals with IBD should be aware of the increased risk if having a malignancy and it is essential to consider primary and secondary prevention measures for neoplasms during daily clinical practice in IBD units.

Hereditary CRC is not modifiable; however, awareness of such family history is critical for early detection and screening (40, 41). 2%-5% of all colon cancers arise from inherited conditions including Lynch syndrome, familial adenomatous polyposis, MUTYH-associated polyposis (41). Close relatives of patients with colorectal cancer are at an increased risk of developing the disease.

Although it largely depends on the region of Turkey and the socioeconomic conditions of individuals, red meat and high-fat foods are significant components of Turkish cuisine. Moreover, with the influence of a more Westernized culture, the consumption of processed foods and red meat has increased, while the intake of fiber-based and vegetable foods (or Mediterranean type diet) has decreased (42, 43). As a result of changes in dietary habits, obesity is also on the rise in the Turkish population (44). With rapid urbanization, physical activity has decreased, and stress factors have increased. Furthermore, more than 20 million individuals, accounting for a prevalence of over 25%, are current tobacco smokers in Turkey, with the majority being young and middle-aged adults (45). These changes in the Turkish population may account for the increased incidence of colorectal cancer (CRC). Although Turkey has implemented a screening program for colorectal cancer after age of 50, involving fecal occult blood tests or colonoscopies, participation in the program is still inadequate (46). Considering the increased risk factors in the Turkish population, preventive measures should be taken, such as enhancing primary healthcare facilities, informing individuals about diet, promoting smoking cessation programs, and increasing affordable sports facilities. Moreover, individuals should be informed and encouraged to participate in regular cancer screening programs. We believe that the first steps are to increase the number of primary healthcare facilities and physicians and to enhance follow-up programs.

## 5 Study strengths and limitations

The current study has several limitations. Firstly, its design as a case-control study means that it may not establish an ideal causal relationship between the variables of interest and CRC due to its observational nature. We observed wide confidence intervals for some variables, which can be due to variability in data. While it is not possible to control for all lifestyle-related factors, we have attempted to include a broad array of such variables, including the type of food participants consume, physical activity, smoking (cigarettes or hookah), alcohol consumption, and sleeping duration. However, we acknowledge that there can be more variables affecting CRC risk. Secondly, there is a potential selection bias, as the study may not have reached the target patients in the population, which is challenging to avoid. Additionally, asking questions related to past events and daily habits introduces recall bias. Lastly, data collection was undertaken in tertiary hospitals, which may not represent the wider population.

The strength of this study lies in its large, case-controlled sample size, which provides a robust dataset for analysis.

## 6 Conclusion

The present study demonstrates that colorectal cancer (CRC) has various risk factors, encompassing dietary, lifestyle, and genetic components. Specifically, this article highlights the modifiable and non-modifiable risk factors. For non-modifiable risk factors, individuals should be aware of their hereditary disposition and be proactive in participating in screening programs. Regarding modifiable risk factors, it is crucial to avoid hookah, cigarette smoking, and alcohol use. Adopting a healthy and balanced diet is essential not only to have a normal body mass index but also to maintain gut health. Individuals should also be vigilant about intestinal inflammatory diseases, as these are associated with an increased risk of CRC. Furthermore, gastrointestinal symptoms, especially chronic changes such as abdominal pain, cramps, diarrhea, constipation, blood in the stool, bloating, nausea/vomiting, and weight loss, should be taken seriously and promptly investigated to facilitate early detection and intervention. The risk factors identified in this study warrant further investigation through longitudinal studies. Additionally, future studies should be planned with a more comprehensive population to closely monitor the trend of these risk factors within the Turkish population.

## Data Availability

The raw data supporting the conclusions of this article will be made available by the authors, without undue reservation.
